# The role of concussion history and biological sex on baseline concussion clinical profile symptoms in adolescent rugby players

**DOI:** 10.1007/s11845-024-03677-7

**Published:** 2024-03-25

**Authors:** Connor McKee, Mark Matthews, Anthony P. Kontos, Alan Rankin, Chris Bleakley

**Affiliations:** 1 Faculty of Life and Health Sciences, Ulster University, York St., Belfast, BT15 1ED Northern Ireland; 2 Department of Orthopaedic Surgery, UPMC Sports Medicine Concussion Program, University of Pittsburgh, Pittsburgh, Pennsylvania, USA; 3 Sports Institute of Northern Ireland, Belfast, Northern Ireland; 4 Sport Medicine NI LTD, Belfast, Northern Ireland

**Keywords:** Anxiety, Depression, Fear-Avoidance

## Abstract

**Background:**

Accurate concussion monitoring requires access to preinjury baseline data. This is particularly important in adolescent athletes who have a high risk of concussion and are prone to prolonged recovery. As Rugby Union is governed by similar laws for men and women, it is also an ideal population to rigorously examine the impact of biological sex on concussion symptoms.

**Aims:**

To evaluate self-reported concussion symptoms at baseline in adolescent rugby union players, and examine if subtype-specific symptoms are affected by concussion history and biological sex.

**Methods:**

Adolescent rugby union players aged 16–18 years were recruited during the 2022–2023 playing season. Participants completed a series of questionnaires covering post-concussion symptoms, concussion clinical profiles, anxiety, depression and fear avoidance behaviours. Independent variables of interest in analysis were biological sex and concussion history.

**Results:**

149 participants (75% male) were included. 42% (63/149) reported at least one previous concussion (average time since concussion: 18.7 months, range 1–72). Adolescents with a concussion history reported significantly higher scores than those with no history, across two clinical profiles (ocular and sleep), concussion symptom severity, and depression, all based on medium effect sizes (SMD 0.3–0.5). Females had significantly higher scores across cognitive/fatigue, ocular and sleep clinical profiles, concussion symptoms, anxiety and depression, each with large effect sizes (SMD > 0.5).

**Conclusions:**

Concussion history and sex are associated with higher baseline scores on specific concussion clinical profile, concussion symptom severity, and anxiety symptoms. These findings highlight the importance of considering baseline differences when interpreting post-injury clinical profile symptoms in adolescent rugby players. (Trial registration: ACTRN12622000931774).

## Introduction

Sport-related concussion is a complex pathophysiological process affecting the brain, induced by traumatic biomechanical forces [1]. Adolescents may be at particularly high risk from concussion in contact and collision sports such as rugby union, ice hockey and American Football [1]. Prospective research indicates that adolescent rugby union has an incidence of 6.1 concussions per 1000 game hours, which is equivalent to 3 concussions per team per season [2]. Clinical recovery after concussion in adolescents is reported to occur within 30 days [1], but in general, recovery post-concussion is more unpredictable in paediatric patients compared to adults [3–8].

Current guidelines [1] suggest that youth athletes participating in high-risk sports should undergo a preseason baseline assessment. This can improve concussion recognition, and inform individualized treatment planning and decision-making on returning a player to training and competitive games. Normative baseline values are increasingly available for adult and collegiate athletes, but are lacking for adolescents, particularly in female sports [9]. It is currently unclear if baseline symptoms in adolescents are affected by concussion history [10–14]. Two large observational studies undertaken in high-school aged ice hockey [11] and American football players [13], consistently reported that athletes with history of multiple prior concussions, had a higher symptom burden at baseline (as quantified by the Post-Concussion Symptom Scale). In contrast, a recent study involving adolescent rugby union players found no significant differences in baseline symptom scores, between those with and without a history of concussion [14].

Baseline symptom reporting is also related to sex. A study of adolescent athletes, found that females were more likely to report baseline symptoms compared to males [12]. A multi-site study across concussion clinics in the United States also found adolescent females to be twice as likely to have a prolonged recovery compared to males, this may relate to biological differences in neck strength, hormonal factors, and psycho-social differences in symptom perception / likelihood of symptom reporting [15]. In a recent systematic review, younger age, female sex, and history of previous concussion were found to be key pre-injury risk factors for prolonged recovery after concussion [16]. Preseason baseline comparisons in Rugby Union players also show significant sex differences, with females reporting a higher number of symptoms and greater symptom severity [17, 18].

Most studies in this field have focused on baseline data on symptom burden and/or neurocognitive function. As concussions can affect multiple systems, baseline data should be multidimensional and include key clinical profiles such as: cognitive, fatigue, anxiety-mood, headache-migraine, ocular, and vestibular constructs [1, 19, 20]. There needs to be a importance of quantifying baseline levels of anxiety and depression [21], with studies suggesting that premorbid psychopathology directly interacts with post-concussion recovery [22].

The aim of this study was to evaluate the role of concussion history and biological sex on overall concussion symptom severity, concussion clinical profile symptoms, as well as anxiety, depression and fear avoidance symptoms at baseline among adolescent rugby union players. As Rugby Union is governed by similar laws for men and women, it is an ideal population to rigorously examine the impact of biological sex on concussion symptoms. It is hypothesized that players with a history of concussion and females would report higher symptoms across all these measures. It is anticipated that there would be an interactive effect for concussion history and biological sex (i.e., female) on these measures.

## Methods

### Study design

This was a cross-sectional study involving adolescents affiliated to Rugby Union playing schools or clubs in the Northern Ireland. The data presented are part of a larger longitudinal study that is tracking recovery after concussion in adolescent rugby players [23].

### Participants

Male and female adolescent rugby union players were recruited from schools and clubs registered in Northern Ireland; some schools and clubs had been previously involved in the Rugby Injury Surveillance in Ulster Schools (RISUS) [2, 24, 25]. To be eligible for inclusion participants must have been aged between 16 and 18 years and injury free at the time of recruitment; they must also have been registered to play rugby union with a school or club for the 2022/23 season.

### Procedures

Ethical approval was granted by the Ulster University Institutional Review Board (REC/14/0060). After providing informed written parental consent and participant assent, participants provided demographic information, injury history and completed the measures (listed below) in the following order: Post-Concussion Symptom Scale (PCSS), Clinical-Profile Screen (CP Screen), Paediatric Fear-Avoidance Behaviours after Traumatic Brain Injury Questionnaire (PFAB-TBI), Generalised Anxiety Disorder Assessment (GAD-7), and the Patient Health Questionnaire (PHQ-9). Baseline testing was completed prior to the start of the 2022–2023 playing season. Researchers were present whilst participants were completing the questionnaires.

Sports-related concussion definition followed the criteria set out by the Consensus Statement on Concussion in Sport; that is, a direct blow to the head, face, neck or elsewhere on the body with an impulsive force transmitted to the head resulting in one or more of the symptoms in one or more of the following clinical domains (which may or may not have involved loss of consciousness): somatic, cognitive, emotional/behavioural and sleep disturbance [26].

## Measures

### Demographics/medical history

This involved completing a demographic questionnaire (age, weight, and height), injury history (previous concussion [yes/no]), and time loss from sport (days) associated with a previous concussion.

#### Concussion symptoms

The Post-Concussion Symptom Scale is a self-reported measure of common concussion symptoms (e.g., headache, dizziness, nausea). The PCSS consists of 22 items rated on a 7-point Likert-type scale ranging from 0 (none) to 6 (severe). Total PCSS symptom severity scores range from 0–132, with higher scores indicating a greater severity of post-concussive symptoms. The scale has high internal consistency [27] in healthy and concussed adolescents, as well as construct and discriminate validity (concussed v non concussed athletes) has been established [27, 28].

####  Concussion clinical profiles

The CP Screen is a self-reported measure of symptoms representing five clinical profiles: cognitive/fatigue, vestibular, ocular, migraine, anxiety/mood; and two modifiers: sleep, neck (34). The CP Screen consists of 29 items rated on a 4-point Likert-type scale ranging from 0 (none) to 3 (severe). Item raw scores are totalled for each profile, with higher scores indicating greater severity of symptoms for that profile. The scale has high internal consistency, as well as construct and discriminate validity [29].

#### Fear/avoidance

The PFAB-TBI is a self-reported measure of fear and avoidance behaviours [30]. The PFAB-TBI includes 16-items related to fear and avoidance behaviour during the past month rated on a 4-point Likert-type scale ranging from 0 (strongly disagree) to 3 (strongly agree). Higher scores indicate greater fear avoidance behaviour (possible range 0–48) [31].

#### Anxiety

The GAD-7 questionnaire is a self-report screen of anxiety symptoms (e.g., nervousness, fear, worry). The GAD-7 includes 7-items examining presence and frequency of anxiety symptoms during the past two weeks rated on a 4-point Likert-type scale ranging from 0 (not at all) to 3 (nearly every day). Total scores range from 0 to 21, with higher scores indicating more severe anxiety symptoms. The GAD-7 has good reliability, as well as criterion, construct, factorial, and procedural validity [32].

#### Depression

The PHQ-9 is a self-report screen for depressive symptoms (e.g., hopelessness, sleep, fatigue, disinterest). The PHQ-9 consists of 9-items that assess the frequency and severity of depressive symptoms during the past two weeks rated on a scale from 0 (not at all) to 3 (nearly every day). Total scores range from 0–27 with higher scores indicating more frequent or severe symptoms. The PHQ-9 has been validated for primary care use [33].

### Statistical analysis

All data were analysed using the Statistical Package of Social Sciences (SPSS) (Version 29; SPSS Inc). Outcomes were summarised using medians (range) for scale variables and frequencies and percentages for nominal variables. We used histograms to examine the frequency distributions of scale variables. All outcome variables were not normally distributed, therefore we used a non-parametric omnibus test (Kruskal-Wallis) followed by pairwise comparisons (Dunn-Bonferroni post hoc tests) across each level of the independent variables: male no history, female no history, male concussion history, female concussion history. As the distribution of data for each group had a similar shape (right skew), we focused on the median scores (rather than the mean ranks). For our primary analysis was used total scores from each questionnaire. We ran separate analyses on each CP Screen factor and modifier scores. The threshold for statistical significance was P < 0.01 for all tests, with Bonferroni adjustments used to reduce the risk of Type 1 error due multiple comparisons. We presented between group differences for concussion history and sex, using standardised mean differences (SMD) and 95% confidence intervals (99% CIs).

### Sample size

The cross-sectional data presented in this study form part of a larger longitudinal investigation tracking recovery after concussion in adolescent rugby players. Therefore, the original sample size was determined based on our longitudinal data, assuming a 20% loss to follow-up throughout the entire study and a 10% prevalence of concussion during a single playing season [2, 24]. Complete details can be found in the published protocol [23]. For the current cross sectional analysis, we needed a minimum sample size of 65 participants per group to test our primary hypothesis (that players with a history of concussion would report higher symptoms). This was based on a medium effect size (SMD 0.5) when comparing independent groups (concussion history vs no history), and alpha and beta levels of 1% and 20% respectively.

## Results

A total of 149 participants, from 10 rugby union teams or clubs, participated in the study (n = 113 male [75.8%], n = 36 female [24.2%]) aged 16 to 18 years (Mean age 17.2yrs ± 0.7). Males were taller and heavier (mean height 180.6 cm ± 6.4, mean weight 83.0 kg ± 15.5) than females (mean height 164.4 cm ± 6.5, mean weight 64.7 kg ± 6.7). Of the 149 participants, 63 (42.3%) reported a history of at least 1 concussion. The average time since last concussion was 18.7 months (range 1–72 months). A total of 47.8% of males (54/113) reported a history of at least 1 concussion, compared to 25.0% of females (9/36).

**Fig. 1:**
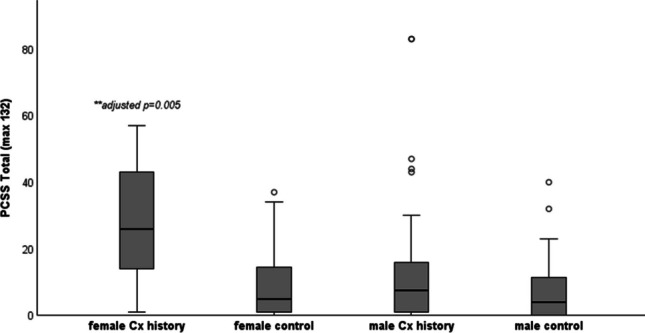
Post-Concussion Symptom Scale (post hoc comparisons). **female Cx history > male no Hx (Dunn Bonferroni post hoc; test statistic 51.1, adjusted p value = 0.005)

**Fig. 2:**
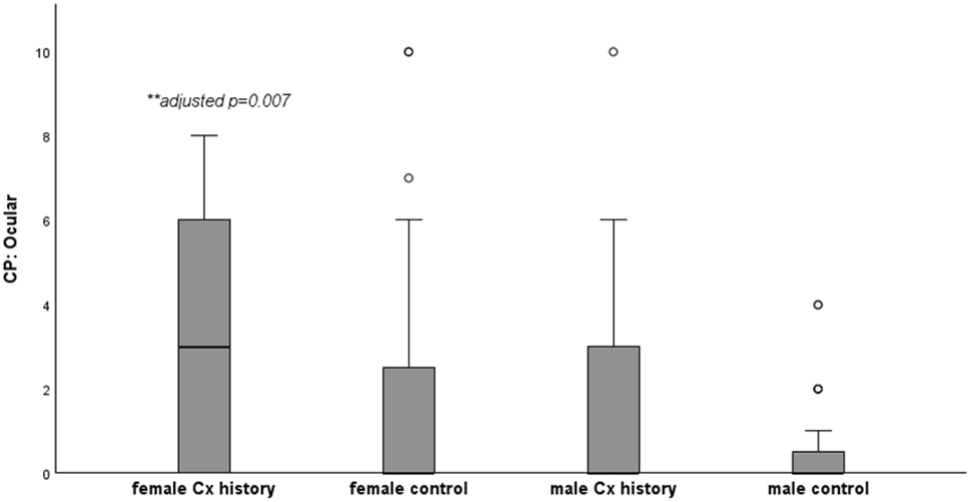
Clinical Profile Screen: Ocular (post hoc comparisons). **female Cx history > male no Hx (Dunn Bonferroni post hoc; test statistic 43.74, adjusted p value = 0.007)

**Fig. 3:**
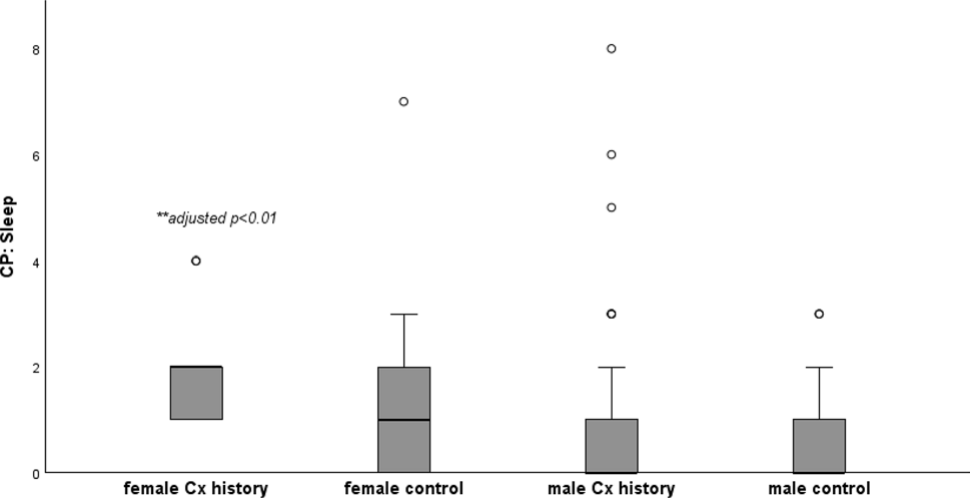
Clinical Profile Screen: Sleep (post hoc comparisons). **female Cx history > male no Hx (Dunn Bonferroni post hoc; test statistic 53.76, adjusted p value = 0.004). *female Cx history > male Cx history (Dunn Bonferroni post hoc test statistic 43.27; adjusted p-value = 0.008)

**Fig. 4:**
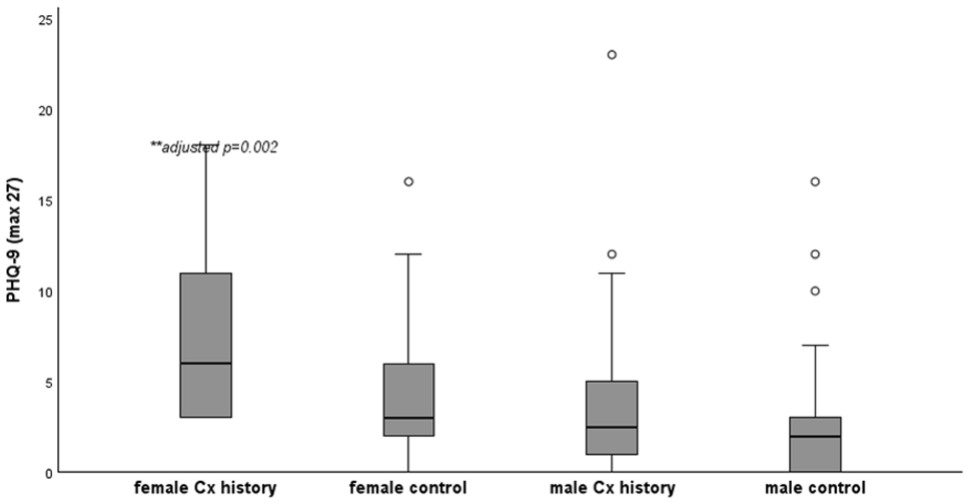
Depression symptoms (PHQ-9; post hoc comparisons). **female Cx history > male no Hx (Dunn Bonferroni post hoc; test statistic 55.23, adjusted p value = 0.002)

Table 1 shows the median (range) scores for each self-reported questionnaire, split by history of concussion and biological sex. Kruskal-Wallis H result supported a statistically significant difference in scores between the independent groups for PCSS (p < 0.01), CP Screen total score (p < 0.001), CP Screen ocular (p < 0.01) and sleep subscales (p < 0.001), PHQ (p < 0.001) and GAD-7 (p < 0.01). The highest median scores for PCSS (26/132), CP Screen (ocular and sleep subscales) and PHQ (6/27), were reported in females with a history of concussion. The boxplots below show the scores for PCSS (Fig. 1), Ocular (Fig. 2), CP Screen (Sleep) (Fig. 3) and PHQ (Fig. 4), split by concussion history and biological sex. The findings from Dunn Bonferroni post-hoc tests are also shown on each figure; the median scores in females with a history of concussion were significantly higher (adjusted p < 0.01) than males with no history for each of these outcomes. For clinical profiles for Sleep, females with a history of concussion also had significantly higher median symptoms (p < 0.01) compared to the males with a history of concussion (Fig. 3). There were no differences for other pairwise comparisons.

**Table 1:** Self-reported questionnaire data split by concussion history and sex (median, range)

	**NO HISTORY**		**CONCUSSION HISTORY**		**BETWEEN GROUP COMPARISON**
	**Male** **(n = 59)**	**Female** **(n = 27)**	**Male** **(n = 54)**	**Female** **(n = 9)**	P-value (omnibus test Kruskal-Wallis)
**PCSS** **(max 132)**	4 (0–40)	5 (0–37)	7.5 (0–83)	26 (1–57)	(χ2(3) = 12.26, p = 0.007
***CP Screen***					
* Total****,**** max 87*	2 (0–27)	4 (0–41)	5.5 (0–54)	16 (2–28)	χ2(3) = 16.78, p = 0.0008
Anxiety Mood	0 (0–4)	0 (0–8)	0 (0–14)	1 (0–7)	χ2(3) = 7.20, p = 0.066
Cognitive Fatigue	0 (0–4)	1 (0–5)	1 (0–8)	2 (0–7)	χ2(3) = 7.20, p = 0.066
Migraine	0 (0–6)	0 (0–14)	0 (0–9)	1 (0–6)	χ2(3) = 10.23, p = 0.02
Ocular	0 (0–4)	0 (0–10)	0 (0–10)	3 (0–8)	χ2(3) = 16.10, p = 0.001
Vestibular	0 (0–6)	0 (0–6)	0 (0–9)	0 (0–6)	χ2(3) = 3.32, p = 0.345
Sleep	0 *(0–3)*	1 (0–7)	0 *(0–8)*	2 *(0–4)*	χ2(3) = 19.10, p = 0.0003
Neck	0 *(0–4)*	0 *(0–2)*	0 *(0–3)*	1 *(0–2)*	χ2(3) = 7.13, p = 0.068
**PHQ (max, 27)**	2 (0–16)	3 (0–16)	2.5 (0–23)	6 (3–18)	χ2(3) = 16.78, p = 0.0008
**GAD-7 (max, 21)**	0 (0–15)	3 (0–12)	1 (0–20)	3 (0–11)	χ2(3) = 12.88, p = 0.005
**PFAB-TBI (max, 48)**	1 (0–26)	0 (0–22)	4 (0–27)	0 (0–16)	χ2(3) = 3.988, p = 0.263

**Fig. 5:**
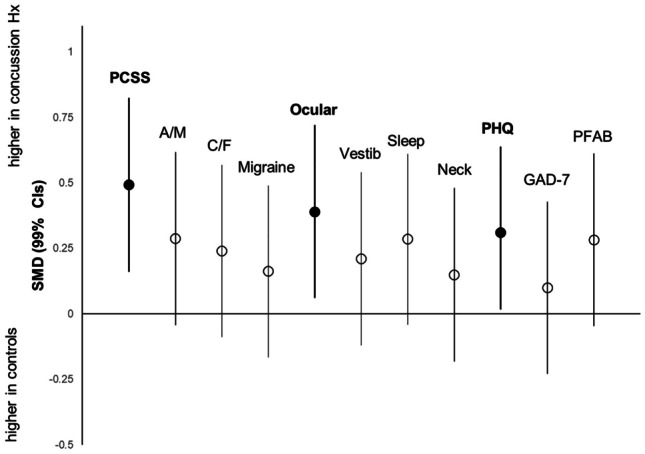
Comparison of self-reported scores (SMD; 99% CI) in adolescents with concussion history versus no history. *Solid black circles represent significant outcomes

**Fig. 6:**
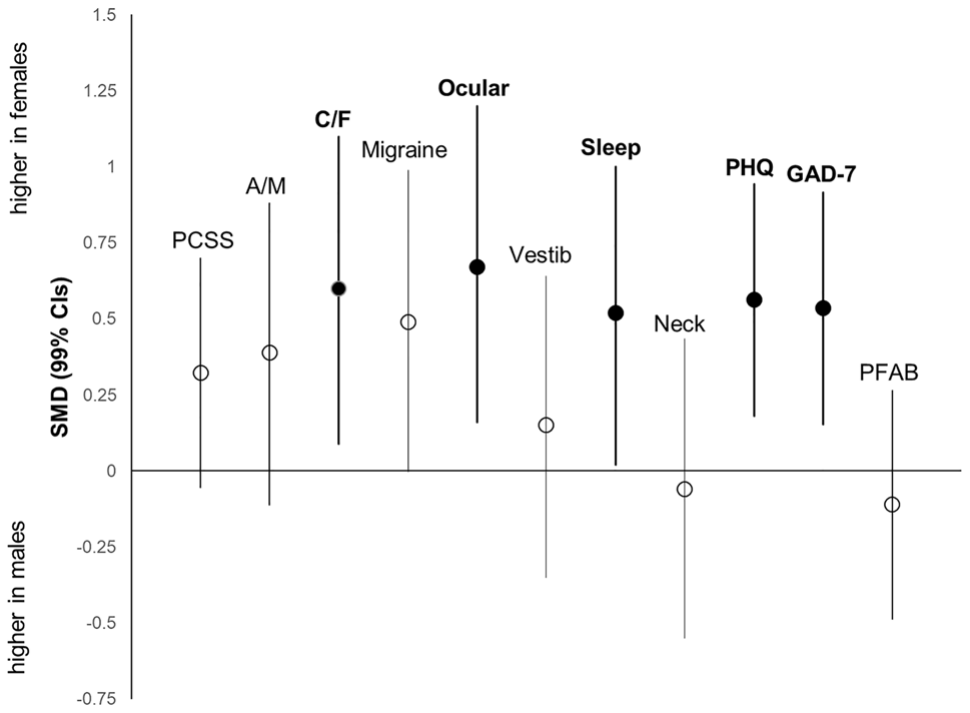
Comparison of self-reported scores (SMD; 99% CI) in adolescent males vs adolescent females. *Solid black circles represent significant outcomes

Figures 5 and 6 show the standardized effect magnitudes (SMD, 99% CIs) associated with concussion history and sex, across all self-reported outcomes. Figure 5 shows that scores were consistently higher in adolescents with a history of concussion compared to controls; the largest differences between groups (depicted by the filled circles) were seen in PCSS (SMD 0.50; 0.16 to 0.82), Ocular (SMD 0.39; 0.06 to 0.72) and PHQ (SMD 0.31; 0.02 to 0.64). Figure 6 shows that the average scores were higher in females across most outcomes, aside from the Neck subscale of CP Screen and PFAB. The largest differences between groups (depicted by filled circles) show that females had significantly higher scores (vs males) across 3 clinical profiles, Cognitive/Fatigue (SMD 0.60; 0.09 to 1.10), Ocular (SMD 0.67; 0.16 to 1.20) and Sleep (SMD 0.52; 0.02 to 1.00), and in depression (PHQ) (SMD 0.56; 0.18 to 0.94) and anxiety (GAD-7) (SMD 0.54; 0.15 to 0.92), all with medium effect sizes.

## Discussion

Sport-related concussions are complex pathophysiological processes affecting the brain, with heterogenous clinical presentations. Accurate concussion monitoring requires access to preinjury baseline data, across multidimensional clinical profiles. There is no clear consensus on the moderating effect that demographic variables (eg. sex and concussion history) have on preseason/baseline data; as Rugby Union is governed by similar laws for men and women, it is an ideal population to rigorously examine this. The aim of this study was to examine self-reported symptoms in adolescent rugby players, across a range of clinical profiles at baseline. This study found concussion history and sex were associated with higher baseline scores for specific clinical profile symptoms. Adolescents with a concussion history reported higher ocular and sleep symptoms, total concussion symptom severity, and depression. Regarding sex, females reported higher cognitive/fatigue, ocular, and sleep clinical profile symptoms, as well as total concussion symptom severity, anxiety, and depression symptoms.

PCSS is one of the most used tools to assess and quantify the severity of concussion symptoms. Consistent with previous research undertaken in groups of adolescent footballers [11, 34], middle school [35] and high school athletes [10, 36], we found that adolescents with previous concussion demonstrated higher symptom scores (on PCSS), compared to those with no prior concussions. In a recent study involving adolescent rugby players, Cosgrave et al. recorded a similar prevalence of previous concussions at baseline (45% vs 42% in the current study), but found no significant difference in baseline symptoms. This divergence may relate to differences in outcome measures (SCAT vs PCSS) and demographics (they exclusively focused on males). We note that in the current study, the median baseline symptom score reported in males with a history of concussion was 7.5, which is largely comparable to previous data (where average scores ranged from 6 to 13) [10, 13, 35].

Separate analyses were run on each Clinical profile screen factor and modifier scores. One of the largest differences was evident in the ocular profile, with higher symptoms reported in athletes with a history of concussion. (SMD 0.39; 0.06 to 0.72). Others report light sensitivity [13] and visual problems [13] as the most commonly endorsed baseline symptoms, with one study finding differences in pupillary light reflex metrics when comparing adolescent rugby players with and without a history of concussion at baseline [37]. Baseline depression scores were higher in adolescents with a history of concussion, based on PHQ-9, but we did not report any differences in anxiety or fear avoidance scores. Earlier retrospective studies in adolescents (13–17 years) found a correlation between concussion history and self-reported depression [38], with previously concussed athletes 2.5 times more likely to report elevated scores (≥ 5) on the PHQ-9 at baseline. The relationship between variables relating to concussion history, baseline symptoms and depression may be complex. For example, it is unclear if the baseline depressive symptoms reported are a reaction to experiencing persistent post-concussion symptoms, or that depressive symptoms increase the likelihood of reporting persisting symptoms.

Statistically significant differences were found between males and females for ocular, cognitive/fatigue and sleep clinical profiles, as well as for depression and anxiety, based on moderate (SMD 0.3–0.5) to large (SMD > 0.5) effect sizes. There was an observed interaction (concussion history*sex) for PCSS, where scores were highest in females with a history of concussion (median scores of 26 points (range 1–57). This value exceeds baseline scores reported in a sample of female adolescents with a history of multiple concussions (18.1 ± 22.1) [39], and is comparable to those reported by adolescents in the early stages (within 7 days) post-concussion [40]. A large cross sectional study in rugby players (average age 19 years) found that sex effects baseline symptom scores, with females reporting significantly higher total SCAT symptoms (p < 0.001) than men [18]. The findings from this study align with previous research showing females report higher baseline symptoms on PCSS [27], ImPACT [41] and for symptoms relating to headache, sleep, vision, and reduced energy [42]. Others have found that females have higher symptom severity on individual clinical profiles of cognitive/fatigue, ocular and anxiety/mood compared with males [43]. These patterns may be due to females being more willing to reporting concussion-related symptoms [44]. Adult studies have also documented differences in concussion prognosis between males and females, with females more likely to present with lingering symptoms [43, 45–48].

The baseline differences recorded for ocular, cognitive/fatigue and sleep clinical profiles, between males and females, provide further evidence that that biological sex is an important covariate to consider for future search in this field. The largest interaction (concussion history*sex) was for the sleep clinical profile, whereby, females with a history of concussion reported significantly more baseline symptoms that males with no history of concussion and males with a history of concussion. This study did not find any interaction effects (concussion history*sex) for PHQ-9 and previous research is inconsistent regarding the influence of sex on depressive outcomes associated with concussion. One study found that among collegiate athletes, women have an 84% increased risk of reporting clinically significant depressive symptoms after sustaining a concussion compared to men [49]. Conversely, a study separate did not reveal any differences in the development of depressive symptoms between male and female athletes following a concussion [50].

Normative baseline data can provide accurate comparisons if individual baseline data are not available [51]. Although baseline data are accessible for adult and collegiate athletic populations, they are lacking for adolescents [9]. In accordance with previous research, this study found that few participants had baseline scores of 0 across the self-reported outcomes assessed [9, 11, 51]. Regardless of concussion history, participants reported sleep issues (PCSS and CP-Screen), anxiety issues (GAD-7 and CP-Screen), fatigue (PCSS, PHQ-9 and CP-Screen), nervousness (GAD-7), drowsiness (PCSS, PHQ-9 and CP-Screen), and difficulty concentrating (PCSS and CP-Screen). This aligns with previous reports [9, 11–13] suggesting that complete symptom resolution post-concussion may not be realistic for all adolescent athletes.

## Limitations

This study had unbalanced comparison groups, particularly for sex, where more males were recruited than females into the study. This affects the generalizability of the findings to the broader population of females. In general, females are under-represented across all sports medicine research, and few have been included in adolescent concussion studies to date [1].

Females with a history of concussion were particularly underrepresented in the current study, and interaction affects (concussion history*sex) were observed for some outcomes, these findings should be interpreted cautiously until it is replicated using a larger, and more balanced representation of females and males.

Measures used in this study were all self-reported, which introduces recall, social desirability, and reference bias into the study. It can only be assumed that participants were honest and accurate in their reporting of symptoms but cannot confirm this assumption. Another limitation of the current study is a lack of temporal standardization across participants. Participants were recruited from a range of schools and clubs at times convenient to their training schedules, which impacted the ability to standardize the time of day that participants completed the study measures. Self-reported symptoms may be affected by diurnal variation, with some symptoms worsening over the course of a day, due to increasing levels of exertion, physical or mental activity [39, 52]. The majority of our data collection occurred in the early afternoon when the effects of diurnal variation are likely to be lower [52]. None the less, the variability in timing could have influenced the responses provided by participants.

## Conclusion

Concussion history and sex each affect baseline symptom reporting in adolescent rugby players. Athletes with a history of concussion reported significantly higher concussion total symptom severity, ocular clinical profile symptoms as well as depression and anxiety symptoms compared to those with no history. Regarding sex, females reported higher ocular, cognitive/fatigue, and sleep clinical profiles symptoms. Females reported higher levels of depression and anxiety symptoms compared to males. The findings also support an interaction effect for sleep clinical profiles, with females with a history of concussion being more likely to report baseline symptoms than males with a history of concussion. Overall, these findings highlight the importance of considering baseline differences when interpreting post-injury clinical profile symptoms in adolescent rugby players.

## Data Availability

The data used and/or analysed during the current study are available from the corresponding author upon reasonable request.
